# Respiratory Variability, Sighing, Anxiety, and Breathing Symptoms in Low- and High-Anxious Music Students Before and After Performing

**DOI:** 10.3389/fpsyg.2020.00303

**Published:** 2020-02-26

**Authors:** Amélie J. A. A. Guyon, Rosamaria Cannavò, Regina K. Studer, Horst Hildebrandt, Brigitta Danuser, Elke Vlemincx, Patrick Gomez

**Affiliations:** ^1^Center for Primary Care and Public Health, University of Lausanne, Lausanne, Switzerland; ^2^School of Applied Psychology, University of Applied Sciences and Arts Northwestern Switzerland, Olten, Switzerland; ^3^Swiss University Centre for Music Physiology, Basel and Zurich Universities of the Arts, Zurich, Switzerland; ^4^School of Biological and Chemical Sciences, Queen Mary University of London, London, United Kingdom

**Keywords:** respiratory variability, sighing, music performance anxiety, breathing symptoms, music students, social self, stress, respiratory psychophysiology

## Abstract

Music performance anxiety (MPA) is a major problem for music students. It is largely unknown whether music students who experience high or low anxiety differ in their respiratory responses to performance situations and whether these co-vary with self-reported anxiety, tension, and breathing symptoms. Affective processes influence dynamic respiratory regulation in ways that are reflected in measures of respiratory variability and sighing. This study had two goals. First, we determined how measures of respiratory variability, sighing, self-reported anxiety, tension, and breathing symptoms vary as a function of the performance situation (practice vs. public performance), performance phase (pre-performance vs. post-performance), and the general MPA level of music students. Second, we analyzed to what extent self-reported anxiety, tension, and breathing symptoms co-vary with the respiratory responses. The participants were 65 university music students. We assessed their anxiety, tension, and breathing symptoms with Likert scales and recorded their respiration with the LifeShirt system during a practice performance and a public performance. For the 10-min periods before and after each performance, we computed number of sighs, coefficients of variation (CVs, a measure of total variability), autocorrelations at one breath lag (ARs(1), a measure of non-random variability) and means of minute ventilation (V’_E_), tidal volume (V_T_), inspiration time (T_I_), and expiration time (T_E_). CVs and sighing were greater whereas AR(1) of V’_E_ was lower in the public session than in the practice session. The effect of the performance situation on CVs and sighing was larger for high-MPA than for low-MPA participants. Higher MPA levels were associated with lower CVs. At the within-individual level, anxiety, tension, and breathing symptoms were associated with deeper and slower breathing, greater CVs, lower AR(1) of V’_E_, and more sighing. We conclude that respiratory variability and sighing are sensitive to the performance situation and to musicians’ general MPA level. Moreover, anxiety, tension, breathing symptoms, and respiratory responses co-vary significantly in the context of music performance situations. Respiratory monitoring can add an important dimension to the understanding of music performance situations and MPA and to the diagnostic and intervention outcome assessments of MPA.

## Introduction

Performing in concerts, competitions, or auditions can be a demanding activity for musicians, especially for those who suffer from music performance anxiety (MPA; Kenny, 2011). MPA has been defined as “the experience of marked and persistent anxious apprehension related to musical performance […] which is manifested through combinations of affective, cognitive, somatic and behavioral symptoms” (Kenny, 2010, p. 433). MPA is a major issue for professionals and students (Fernholz et al., 2019).

Music performance situations can be apprehended as social-evaluative stressors. According to the self-preservation theory, human beings are motivated to preserve their social self, i.e. their status and worth within a social group (Kemeny, 2009). Several factors can make the experience of performing feel threatening to musicians. These include that a high performance quality is an important goal to the musicians’ self-identity, that the performance requires the display of high-level skills, that the performance is evaluated implicitly or explicitly by others and that there are elements that are uncontrollable and unpredictable (e.g. performance of other musicians; size, composition, and behavior of the audience). In line with this view, self-reported anxiety, distress, nervousness, bodily complaints, and negative perceptions are in most musicians greater before and during public performances compared to practice (e.g. Craske and Craig, 1984; Fancourt et al., 2015). Compared to practice, public performances are also associated in most musicians with neuroimmunoendocrine changes that can be interpreted as signs of enhanced physiological arousal (e.g. Craske and Craig, 1984; Kusserow et al., 2012; Aufegger and Wasley, 2018).

Only four studies have investigated differences in performance-related psychophysiological activation between musicians reporting relatively higher or lower MPA during practice and public performances (Craske and Craig, 1984; Fredrikson and Gunnarsson, 1992; Studer et al., 2012, 2014). No significant differences were found in endocrine measures, skin conductance, and heart rate as a function of musicians’ MPA level. In contrast, MPA was significantly associated with changes in the partial pressure of end-tidal carbon dioxide (PetCO_2_) and minute ventilation (i.e. the volume of air inhaled per minute).

Respiratory regulation is characterized by complex variability (Pool, 1989) with random (i.e. time and volume parameters of subsequent breaths are uncorrelated) and non-random (i.e. time and volume parameters of subsequent breaths are correlated; Bruce and Daubenspeck, 1995) components. Their dynamic balance is crucial in ensuring that blood gas levels, and in particular partial pressure of CO_2_, remain within tightly set limits (Vlemincx et al., 2013a). A healthy respiration balances random variability warranting flexibility in response to internal and external demands and non-random variability ensuring stability (Bruce, 1996).

Sighing appears to function as a psychophysiological reset mechanism restoring homeostatic balance when this has been compromised (e.g. Soltysik and Jelen, 2005; Vlemincx et al., 2009, 2010a, 2016). Sighing restores lung compliance and gas exchange efficiency and reduces hypoxia and hypercapnia (Cherniack et al., 1981). Sighing was shown to reset non-random respiratory variability when breathing becomes increasingly random, to be followed by increased stress relief and decreased muscle tension (e.g. Baldwin et al., 2004; Vlemincx et al., 2010b), and to be enhanced during dyspnea relief (Vlemincx et al., 2018).

Research suggests that total respiratory variability (quantified by the coefficient of variation or *SD*), non-random respiratory variability (quantified by autocorrelation at one breath lag), and sighing frequency are sensitive to emotional, cognitive, and behavioral demands. In healthy individuals, stressful and aversive situations such as a mental arithmetic task or viewing unpleasant high-arousal pictures induce an increase in total respiratory variability, a decrease in non-random respiratory variability, and more frequent sighing compared to emotionally neutral conditions (e.g. Keefe and Block, 1982; Boiten, 1998; Rainville et al., 2006; Vlemincx et al., 2011, 2015; but see Van Diest et al., 2006).

Compared to healthy controls, individuals reporting relatively high anxiety sensitivity (Vlemincx et al., 2017), suffering from chronic anxiety (Tobin et al., 1983a, b), and from anxiety disorders or phobias (Abelson et al., 2001; Wilhelm et al., 2001; Alpers et al., 2005; Ritz et al., 2009; but see Blechert et al., 2007; Pfaltz et al., 2009) exhibit more frequent sighing than healthy individuals under rest or stress conditions. The few studies that have investigated the relationships between personal characteristics such as trait anxiety and respiratory variability provide a less coherent picture (Wilhelm et al., 2001; Van den Wittenboer et al., 2003; Alpers et al., 2005; Van Diest et al., 2006; Pfaltz et al., 2009). For instance, whereas Wilhelm et al. (2001) found greater total respiratory variability in panic disorder patients than healthy controls, Pfaltz et al. (2009) did not replicate this finding. How respiratory variability and sighing may depend on the interaction between situational factors and personal characteristics is poorly known.

Response system coherence in emotion refers to the extent to which different response systems (i.e. experiential, physiological, behavioral) covary during emotion. Response system coherence has been postulated by several emotion theories (Ekman, 1992; Levenson, 1994) though empirical support is mixed (Evers et al., 2014).

In the context of music performance situations, most studies have failed to show significant response coherence between self-reported experience and physiological reactivity (Craske and Craig, 1984; Fredrikson and Gunnarsson, 1992; Gill et al., 2006; Studer et al., 2012; Wasley et al., 2012; Pilger et al., 2014; Fancourt et al., 2015). In these studies, the authors evaluated response system coherence at the between-person level by computing correlations; response system coherence at the within-person level was never investigated. Compared to analysis of between-person associations, analysis of within-person associations should be more sensitive to detecting response system coherence because sources of between-individual variance are minimized in within-participants designs (Mauss et al., 2005). Moreover, within-person associations would conceptually denote more closely response system coherence as implied by emotion theories than between-person associations (Mauss et al., 2005).

Evers et al. (2014) have proposed the dual-process framework of emotion response coherence to reconcile inconsistent findings regarding response system coherence in emotion. This account distinguishes between two main systems, an automatic system (relatively unconscious, fast, and effortless) and a reflective system (relatively conscious, deliberate, and effortful). According to this framework, response coherence should be maximal within each system and minimal across the two systems. Self-reported experiences represent relatively reflective responses. On the contrary, (most) physiological responses (e.g. blood pressure) constitute relatively automatic responses, which are relatively difficult to control and of which people are relatively unaware. Respiration is situated at the intersection of automatic functioning and voluntary control. Compared to most other physiological systems, we might then expect relatively good response coherence between self-reported experience and breathing parameters.

The present study is part of a research project on the cardiorespiratory responses in university music students when performing with and without an audience present. Previous publications based on data collected in this project have addressed questions regarding the mean cardiorespiratory responses before, during, and after the practice and public performances (Studer et al., 2012, 2014). Here, our investigations into the respiratory psychophysiology of music performance situations and MPA focus on indices of respiratory variability and sighing and on the coupling of these measures with self-reported anxiety, tension, and breathing symptoms.

We had two goals. The first goal was to investigate whether respiratory measures, self-reported affective state (anxiety and tension) and breathing symptoms (shortness of breath and difficulty in breathing deeply enough) vary as a function of the performance situation and of the general MPA level of the music students. With regard to the respiratory measures, our focus was on the less established measures of respiratory variability and sighing, for which we formulated specific hypotheses. We also analyzed means of respiratory variables but did not formulate any specific hypotheses given their secondary role in this study. With regard to the performance situation, we considered the effects of two factors: session and phase. Session refers to whether the participants performed without an audience present (practice session) or with an audience present (public session). Phase refers to whether the measurements took place immediately before the performance (pre-performance phase) or immediately after the performance (post-performance phase). We tested four sets of hypotheses. Hypothesis 1 was that compared to the practice session, the public session would be more threatening to the social self and, therefore, would be associated with greater self-reported anxiety and tension, more breathing symptoms, higher total respiratory variability, lower non-random respiratory variability, and more sighing. Hypothesis 2 was that compared to the post-performance phase, the pre-performance phase would be more threatening to the social self and, therefore, would be associated with significantly greater self-reported anxiety and tension, more breathing symptoms, higher total respiratory variability, lower non-random respiratory variability, and more sighing. Hypothesis 3 was that the session effect would be significantly larger during the pre-performance phase than the post-performance phase and that the phase effect would be significantly larger during the public session than the practice session. Hypothesis 4 was that the public session would be more threatening to the social self of participants reporting relatively higher than lower MPA, and thus, the session effect would be larger for participants reporting relatively higher than lower MPA.

The second goal was to analyze to what extent self-reported affective state and breathing symptoms co-vary with the respiratory responses at the between- and within-person levels. Hypothesis 5 was that self-reported anxiety and tension would be significantly associated with higher total respiratory variability, lower non-random respiratory variability, and more sighing. Hypothesis 6 was that self-reported breathing symptoms would be significantly associated with higher total respiratory variability, lower non-random respiratory variability, and more sighing.

## Materials and Methods

### Participants

The participants were 27 males and 38 females students enrolled in Swiss university music schools (see Table 1). All participants gave written informed consent. The materials can be obtained from the corresponding author.

**TABLE 1:** Participants’ characteristics.

	*N*	%	Mean (*SD*)	Range
Sample size	65			
**Gender**				
Men	27	41.5%		
Women	38	58.5%		
**Hormonal contraception**				
Yes	18	47.4%		
No	20	52.6%		
Age (years)			23.1 (3.4)	16–30
BMI (kg/m^2^)			21.5 (2.6)	16.1–29.6
MPA			46.5 (11.2)	24–74
TA			41.0 (10.5)	23–69
**Instrument group**				
Wind instrumentalists/singers	30	46.2%		
Other instrumentalists	35	53.8%		

*BMI, body mass index; MPA, music performance anxiety; TA, trait anxiety. MPA and TA scores can range from 20 (no anxiety) to 80 (severe anxiety). Wind instrumentalists/singers included woodwind instruments (flute, clarinet, and bassoon, 20%), brass instruments (trombone and trumpet, 7.7%), and voice (18.5%). Other instrumentalists included keyboard (piano and accordion, 20%), bowed string (violin, cello and double bass, 21.5%), plucked string (harp and guitar, 7.7%) and percussions (4.6%). Players of flute, clarinet, bassoon, trombone, trumpet, violin, double bass, percussions, and singers performed while standing. Players of piano, accordion, cello, harp, and guitar performed while sitting.*

### Procedure

We tested participants in three sessions: familiarization, practice, and public, in this order and separated by approximately 1 week.

#### Familiarization Session

First, the experimenter informed the participants about the study and obtained written consent from them. Then, the Lifeshirt system was applied, a respiratory volume calibration was carried out, and the participants were given a few minutes to play their instruments. After putting on the nasal cannula, the participants filled in questionnaires assessing their usual MPA level and their TA and sat alone for 10 min. We did not analyze the respiratory data collected during this session, as this was not a goal of the present study.

#### Practice and Public Sessions

These sessions were identical except for the fact that the participants performed without audience in the practice session and in front of an audience of 8 to 10 persons in the public session. We told the participants that among the audience there would be two experts who would evaluate their performance and that the public performance would be audio recorded. The participants chose moderately difficult pieces lasting 6 to 10 min, which were performed in both sessions.

After putting on and calibrating the LifeShirt, warming up, and putting on the nasal cannula, the participants sat alone for a 10-min period at the end of which they rated their affective state and breathing symptoms. Following the removal of the cannula, they were accompanied to a concert room to perform. After coming back to the preparation room and putting on the cannula, they rated their affective state and breathing symptoms during the performance. Finally, they were left alone for a 10-min period at the end of which they rated their affective state and breathing symptoms. The participants received 140 Swiss francs for their participation.

### Measurements

#### Respiratory Measures

Respiratory parameters were acquired with the LifeShirt system (Vivometrics, Inc.), a vest incorporating a respiratory inductive plethysmograph (Sackner et al., 1989). We investigated inspiration time (T_I_ in s), expiration time (T_E_ in s), tidal volume (V_T_ in ml), and minute ventilation (V’_E_ in l/min).

For each of the four measures V’_E_, V_T_, T_I_, and T_E_, we computed the mean, the coefficient of variation (CV), and the autocorrelation at one breath lag (AR(1)). CV represents a measure of total variability and is calculated as SD/mean^∗^100. AR(1) is a measure of correlated respiratory variability and indicates the correlation between a series of consecutive breaths and the same series shifted one breath (Vlemincx et al., 2015).

We computed means, CVs, ARs(1), and number of sighs for the 10-min periods practice pre-performance, practice post-performance, public pre-performance, and public post-performance. We computed CV including and excluding sighs and ARs(1) including sighs (Vlemincx et al., 2015). We defined a sigh as a breath having V_T_ exceeding at least twice the participant’s V_T_ mean of the corresponding 10-min period (Vlemincx et al., 2013a). We did not analyze the respiratory responses during the performance phase because of the large respiratory variability generated by playing different instruments.

#### Self-Reported Measures

Participants rated their anxiety, tension, shortness of breath, and difficulty in breathing deeply enough using 11-point Likert scales ranging from 1 “not at all” to 11 “extremely.”

#### Motion

An accelerometer attached to the LifeShirt measured motion along the x-axis and y-axis, producing a value between 0 (no movement) and 50 (running very fast).

#### Music Performance Anxiety (MPA)

We assessed the usual MPA level with the state scale of the State-Trait Anxiety Inventory (STAI; Spielberger, 1983), which consists of 20 items, e.g. “I am tense,” rated on a four-point Likert scale (1 “not at all” to 4 “very much so”). The score ranges from 20 (no anxiety) to 80 (severe anxiety). We refer to this score as MPA. Because anxiety depends on the performance setting (Cox and Kenardy, 1993), we asked the students to indicate how they had felt before recent solo performances (see Widmer et al., 1997; Nielsen et al., 2018). Cronbach’s alpha in this study was 0.92.

#### Trait Anxiety (TA)

TA was measured using the trait scale of the STAI (Spielberger, 1983), which consists of 20 items, e.g. “I feel inadequate,” rated on a four-point Likert scale (1 “almost never” to 4 “almost always”). The score ranges from 20 (no anxiety) to 80 (severe anxiety). Cronbach’s alpha in this study was 0.91. The correlation between MPA and TA was 0.42.

#### Statistical Analyses

We performed the statistical analyses using Stata 15.0 (Stata Statistical Software, StataCorp LP, College Station, TX, United States). We used an alpha level of 0.05. To approximate normal distribution, we log-transformed scores of the respiratory variables except AR(1) of V’_E_ and AR(1) of T_E_.

First, we performed factor analyses of the respiratory measures (CVs, ARs(1), and means, separately) and the self-reported measures (affective state and breathing symptoms, separately) to determine whether these variables could be considered repeated measures of the same latent variables and thus be tested together in one model. The criteria were that the factor analysis had to identify only one factor with eigenvalue larger than one, that the variables had to load on the same factor and that the Cronbach’s alpha had to be larger than 0.80.

Respiratory CVs, ARs(1), and means were subjected to multilevel mixed-effects linear regression analyses, with restricted maximum likelihood estimation and heterogeneous residual variance structure. Number of sighs was fitted with multilevel mixed-effects negative binomial regression, with the fixed-effects coefficients reported as incidence-rate ratios (IRR = exp(β)). Because the low number of response categories prevented a Gaussian approximation, the self-reported measures were subjected to multilevel mixed-effects ordered logistic regression, with the fixed-effects coefficients reported as odds ratios (OR = exp(β)).

### Effects of Session, Phase, and MPA

We fitted two models. In model 1, we tested fixed main effects of the predictors of interest session (practice vs. public), phase (pre-performance vs. post-performance), and MPA. In model 2, we added to model 1 the interactions session × phase, session × MPA, and phase × MPA. Control variables were TA, age, BMI, motion, gender (men vs. women), hormonal contraception (naturally cycling vs. using hormonal contraception), and instrument group (wind instrumentalists/singers vs. other instrumentalists). In model 2, we also added the interactions session × TA and phase × TA because of the relatedness between MPA and TA.

### Relationships Between Self-Reported Measures and Respiratory Measures

We decomposed anxiety, tension and breathing symptoms into their between-person and within-person components and fitted for each respiratory measure multilevel mixed-effects models with these components as main predictors and the control variables TA, age, BMI, motion, gender, hormonal contraception, and instrument group.

## Results

### Effects of Session, Phase, and MPA

Tables 2, 3 give the means and *SD*s of the respiratory measures and self-reported measures, respectively. Tables 4–6 give the results of the analyses.

**TABLE 2:** Means (*SD*s) of the respiratory parameters during the pre- and post-performance phases of the practice session and of the public session.

		Practice session		Public session	
			
		Pre-performance	Post-performance	Pre-performance	Post-performance
CVs including sighs	V’_E_	33.2 (12.1)	36.3 (16.8)	38.2 (15.5)	39.7 (16.7)
	V_T_	40.1 (15.4)	42.2 (15.8)	45.5 (15.3)	44.3 (17.9)
	T_I_	25.1 (14.1)	24.7 (13.2)	30.4 (16.9)	26.0 (12.9)
	T_E_	31.4 (14.2)	32.0 (14.7)	35.5 (16.8)	34.0 (14.1)
CVs excluding sighs	V’_E_	28.9 (9.5)	31.0 (11.8)	31.0 (9.5)	33.9 (13.1)
	V_T_	26.8 (8.2)	27.6 (8.3)	30.8 (10.8)	29.0 (8.4)
	T_I_	22.3 (14.2)	21.7 (13.0)	27.2 (17.1)	23.1 (12.8)
	T_E_	28.2 (13.7)	27.9 (13.2)	32.2 (15.7)	30.3 (11.8)
ARs(1)	V’_E_	0.19 (0.15)	0.21 (0.16)	0.15 (0.14)	0.18 (0.14)
	V_T_	0.11 (0.14)	0.10 (0.14)	0.12 (0.13)	0.11 (0.13)
	T_I_	0.13 (0.13)	0.06 (0.12)	0.12 (0.17)	0.07 (0.15)
	T_E_	0.20 (0.14)	0.20 (0.13)	0.22 (0.15)	0.15 (0.15)
Means	V’_E_	5.65 (2.22)	5.47 (2.16)	6.30 (2.64)	5.86 (2.52)
	V_T_	353 (147)	340 (154)	410 (172)	350 (151)
	T_I_	1.44 (0.26)	1.40 (0.25)	1.53 (0.33)	1.33 (0.20)
	T_E_	2.42 (0.59)	2.42 (0.56)	2.57 (0.73)	2.39 (0.59)
Number of sighs		3.3 (2.1)	3.5 (2.7)	4.9 (3.4)	4.2 (3.2)

*CVs, coefficients of variation; ARs(1), autocorrelations at one breath lag; V’_*E*_, minute ventilation; V_*T*_, tidal volume; T_*I*_, inspiratory time; T_*E*_, expiratory time; units for means are as follows: V’_*E*_, l/min; V_*T*_, ml; T_*I*_, s; T_*E*_, s; CVs and ARs(1) are dimensionless.*

**TABLE 3:** Means (*SD*s) of the self-reported measures anxiety, tension, shortness of breath, and difficulty in breathing deeply enough during the pre- and post-performance phases of the practice session and of the public session.

	Practice session		Public session	
		
	Pre-performance	Post-performance	Pre-performance	Post-performance
Anxiety	1.8 (1.3)	1.2 (0.5)	3.1 (2.2)	1.4 (0.8)
Tension	1.9 (1.3)	1.3 (0.6)	3.3 (2.1)	1.6 (1.0)
Shortness of breath	1.4 (0.6)	1.1 (0.3)	2.0 (1.4)	1.2 (0.6)
Difficulty in breathing deeply enough	1.5 (0.8)	1.2 (0.7)	2.2 (1.5)	1.4 (0.8)

**TABLE 4:** Estimated mixed-effects linear regression models for CVs including and excluding sighs, and for the four ARs(1).

	CV including sighs		CV excluding sighs		AR(1) of V’_E_		AR(1) of V_T_		AR(1) of T_I_		AR(1) of T_E_	
						
	Coeff.	*SE*	Coeff.	*SE*	Coeff.	*SE*	Coeff.	*SE*	Coeff.	*SE*	Coeff.	*SE*
**Main effects**												
Session	0.071*	0.029	0.061*	0.026	−0.032*	0.016	0.009	0.014	**−**0.001	0.016	**−**0.005	0.016
Phase	0.030	0.027	0.020	0.025	0.027	0.016	**−**0.010	0.014	−0.053***	0.015	−0.037*	0.016
MPA	−0.008*	0.003	−0.007*	0.003	0.002	0.001	**−**0.000	0.001	**−**0.000	0.001	0.000	0.001
TA	0.009*	0.004	0.005	0.003	**−**0.002	0.001	**−**0.001	0.001	**−**0.001	0.001	**−**0.000	0.001
Age	0.018	0.011	0.022*	0.010	**−**0.005	0.004	0.001	0.003	0.003	0.003	0.001	0.004
Gender	0.003	0.087	0.022	0.076	0.029	0.035	0.006	0.027	−0.059**	0.022	**−**0.035	0.031
Hormonal contraception	**−**0.118	0.088	−0.171*	0.003	0.015	0.035	**−**0.006	0.027	−0.048*	0.022	**−**0.017	0.031
BMI	0.032*	0.014	0.015	0.012	**−**0.002	0.006	**−**0.003	0.004	**−**0.004	0.003	**−**0.001	0.005
Instrument group	**−**0.024	0.072	0.000	0.063	0.024	0.029	**−**0.011	0.022	0.015	0.018	**−**0.010	0.026
Motion	0.561***	0.148	0.470**	0.149	0.019	0.065	0.020	0.055	**−**0.026	0.057	**−**0.094	0.064
**Interactions**												
Session × Phase	**−**0.062	0.054	**−**0.046	0.048	0.003	0.031	0.003	0.027	0.014	0.032	−0.070*	0.031
Session × MPA	0.006*	0.003	0.006*	0.002	**−**0.001	0.001	**−**0.000	0.001	**−**0.002	0.002	**−**0.001	0.001
Phase × MPA	**−**0.004	0.003	**−**0.002	0.002	0.000	0.001	0.003*	0.001	0.002	0.001	0.001	0.001
Session × TA	**−**0.005	0.003	**−**0.005	0.003	0.003*	0.002	0.003	0.001	0.002	0.002	**−**0.001	0.002
Phase × TA	0.003	0.003	0.002	0.003	**−**0.003	0.002	**−**0.002	0.001	**−**0.001	0.002	0.002	0.002

CV, coefficient of variation; AR(1), autocorrelation at one breath lag; V’_*E*_, minute ventilation; V_*T*_, tidal volume; T_*I*_, inspiratory time; T_*E*_, expiratory time; MPA, music performance anxiety; TA, trait anxiety; BMI, body mass index; Coeff. = estimated coefficient; *SE* = standard error. Reference categories for categorical predictors are as follows: session: practice; phase: pre-performance; gender: women; hormonal contraception: naturally cycling women; instrument group: other instrumentalists. For continuous predictors, coefficients express the change in the outcome measure per unit of the corresponding scale. Units are as follows: MPA and TA: 1 point on the corresponding scales; age: 1 year; BMI: 1 kg/m^2^; motion: 1 point. Statistically significant results are marked in bold. *** *p* < 0.001, ** *p* < 0.01, * p < 0.05.

**TABLE 5:** Estimated mixed-effects regression models for number of sighs and respiratory means.

	Number of sighs		Mean of V’_E_ and V_T_		Mean of T_I_		Mean of T_E_	
				
	IRR	95% CI	Coeff.	*SE*	Coeff.	*SE*	Coeff.	*SE*
**Main effects**								
Session	1.24**	1.08–1.43	0.137***	0.035	0.001	0.013	0.004	0.015
Phase	0.98	0.86–1.11	−0.144***	0.033	−0.044***	0.010	**−**0.014	0.015
MPA	1.00	0.98–1.01	0.022	0.012	0.000	0.002	**−**0.002	0.003
TA	1.01	1.00–1.03	**−**0.016	0.013	**−**0.001	0.002	0.000	0.003
Age	1.01	0.97–1.05	**−**0.014	0.039	0.006	0.006	0.011	0.009
Gender	0.92	0.65–1.29	0.52	0.30	0.010	0.049	**−**0.026	0.068
Hormonal contraception	0.76	0.53–1.07	0.59	0.30	**−**0.011	0.050	**−**0.026	0.068
BMI	1.05	1.00–1.11	0.076	0.049	0.016*	0.008	0.017	0.011
Instrument group	0.97	0.73–1.29	**−**0.020	0.251	0.074	0.041	0.005	0.057
Motion	3.06***	1.59–5.90	0.708**	0.229	**−**0.003	0.055	**−**0.054	0.063
**Interactions**								
Session × Phase	0.79	0.61–1.02	−0.149*	0.064	−0.097***	0.026	**−**0.062	0.034
Session × MPA	1.01*	1.00–1.03	**−**0.005	0.003	**−**0.000	0.001	0.001	0.001
Phase × MPA	1.00	0.98–1.01	**−**0.001	0.003	0.002**	0.001	0.003*	0.001
Session × TA	0.99	0.98–1.01	0.006	0.004	0.000	0.001	0.000	0.002
Phase × TA	1.00	0.99–1.02	**−**0.005	0.003	**−**0.000	0.001	0.001	0.001

V’_*E*_, minute ventilation; V_*T*_, tidal volume; T_*I*_, inspiratory time; T_*E*_, expiratory time; MPA, music performance anxiety; TA, trait anxiety; BMI, body mass index; Coeff., estimated coefficient; *SE*, standard error; IRR, incidence-rate ratio; CI, confidence interval. Reference categories for categorical predictors are as follows: session: practice; phase: pre-performance; gender: women; hormonal contraception: naturally cycling women; instrument group: other instrumentalists. For continuous predictors, coefficients express the change in the outcome measure per unit of the corresponding scale. Units are as follows: MPA and TA: 1 point on the corresponding scales; age: 1 year; BMI: 1 kg/m^2^; motion: 1 point. Statistically significant results are marked in bold. *** *p* < 0.001, ** *p* < 0.01, * p < 0.05.

**TABLE 6:** Estimated mixed-effects ordered logistic regression models for anxiety and tension, and for breathing symptoms.

	Anxiety and tension		Breathing symptoms	
		
	OR	95% CI	OR	95% CI
**Main effects**				
Session	10.83***	5.13–22.88	8.91***	3.16–25.13
Phase	0.03***	0.01–0.06	0.03***	0.01–0.10
MPA	1.07*	1.01–1.13	1.08	0.99–1.17
TA	1.07*	1.00–1.14	1.10*	1.00–1.21
Age	1.12	0.92–1.35	0.83	0.63–1.11
Gender	1.50	0.34–6.64	0.92	0.11–7.90
Hormonal contraception	2.07	0.47–9.16	2.71	0.33–22.33
BMI	1.04	0.82–1.32	0.99	0.70–1.40
Instrument group	1.20	0.35–4.07	3.45	0.57–20.67
Motion	1.74	0.11–26.66	4.59	0.09–237.23
**Interactions**				
Session × Phase	0.09***	0.03–0.34	0.39	0.06–2.63
Session × MPA	1.09**	1.02–1.16	1.10*	1.00–1.21
Phase × MPA	1.06	1.00–1.13	1.03	0.94–1.13
Session × TA	0.97	0.91–1.03	0.98	0.89–1.08
Phase × TA	0.98	0.92–1.04	1.00	0.90–1.10

MPA, music performance anxiety; TA, trait anxiety; BMI, body mass index; OR, odds ratio; CI, confidence interval. Reference categories for categorical predictors are as follows: session: practice; phase: pre-performance; gender: women; hormonal contraception: naturally cycling women; instrument group: other instrumentalists. For continuous predictors, ORs are per change of one unit of the corresponding scale. Units are as follows: MPA and TA: 1 point on the corresponding scales; age: 1 year; BMI: 1 kg/m^2^; motion: 1 point. Statistically significant results are marked in bold. *** *p* < 0.001, ** *p* < 0.01, * p < 0.05.

#### Coefficients of Variation (CVs)

The factor analyses of the CVs including and excluding sighs revealed that these variables could be tested together as repeated measures of the same latent variables “CV including sighs” and “CV excluding sighs,” respectively.

**FIGURE 1:**
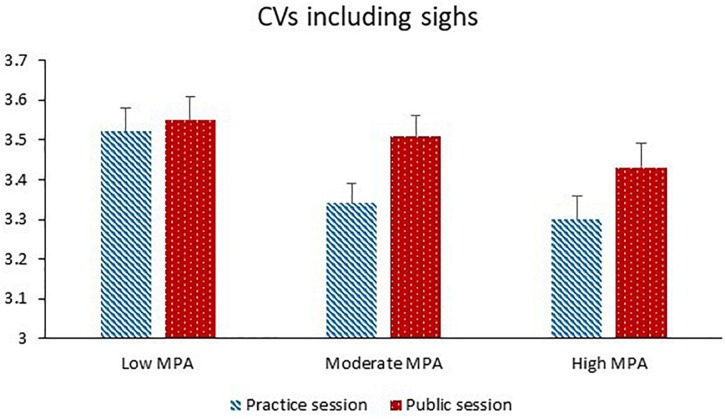
Coefficients of variation (CVs) including sighs (dimensionless) during the practice session and the public session for participants with low MPA (*M* = 34.3, *SD* = 5.0, *n* = 22), moderate MPA (*M* = 46.3, *SD* = 3.0, *n* = 21), and high MPA (*M* = 58.7, *SD* = 6.1, *n* = 22).

Coefficients of variation including sighs was significantly higher during the public session than the practice session. Higher levels of MPA were significantly associated with lower CV including sighs, whereas higher levels of TA were significantly associated with higher CV including sighs. Both higher BMI and higher motion were significantly associated with higher CV including sighs. Session and MPA significantly interacted with each other (see Figure 1). The session effect was significant for MPA levels higher than 45 (*p*s = 0.001–0.021). The MPA effect was significant for the practice session (*p* = 0.004) and non-significant for the public session (*p* = 0.29).

**FIGURE 2:**
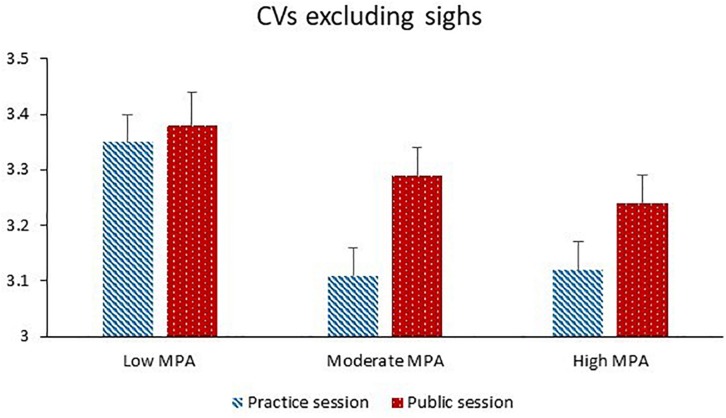
Coefficients of variation excluding sighs (dimensionless) during the practice session and the public session for participants with low MPA (*M* = 34.3, *SD* = 5.0, *n* = 22), moderate MPA (*M* = 46.3, *SD* = 3.0, *n* = 21), and high MPA (*M* = 58.7, *SD* = 6.1, *n* = 22).

Coefficients of variation excluding sighs was significantly higher during the public session than the practice session. Higher levels of MPA were significantly associated with lower CV excluding sighs. Compared to naturally cycling women, women taking hormonal contraceptives had significantly lower CV excluding sighs. Increasing age and increasing motion were both significantly associated with higher CV excluding sighs. Session and MPA significantly interacted with each other (see Figure 2). The session effect was significant for MPA levels higher than 45 (*p*s = 0.001–0.029). The MPA effect was significant for the practice session (*p* = 0.001) and non-significant for the public session (*p* = 0.36).

#### Autocorrelations at One Breath Lag (ARs(1))

The factor analysis of the four ARs(1) revealed that these variables had to be analyzed separately. AR(1) of V’_E_ was significantly lower during the public session than the practice session. Session and TA significantly interacted with each other. The session effect was significant for TA levels lower than 40 (*p*s = 0.005–0.033). The TA effect was not significant for either the practice session (*p* = 0.067) or the public session (*p* = 0.96).

For AR(1) of V_T_, phase and MPA significantly interacted with each other. AR(1) of V_T_ was lower during the post-performance phase than the pre-performance phase for MPA levels lower than 35 (*p*s = 0.035–0.047). The MPA effect was not significant for either the pre-performance phase (*p* = 0.31) or the post-performance phase (*p* = 0.80).

AR(1) of T_I_ was significantly lower during the post-performance phase than the pre-performance phase. Women exhibited significantly higher AR(1) of T_I_ than men, and naturally cycling women had significantly higher AR(1) of T_I_ than women using hormonal contraceptives.

AR(1) of T_E_ was significantly lower during the post-performance phase than the pre-performance phase. Session and phase interacted significantly with each other. The phase effect was not significant for the practice session (*p* = 0.87) but was significant for the public session (*p* = 0.001). The session effect was not significant for either the pre-performance phase (*p* = 0.20) or the post-performance phase (*p* = 0.062).

#### Number of Sighs

**FIGURE 3:**
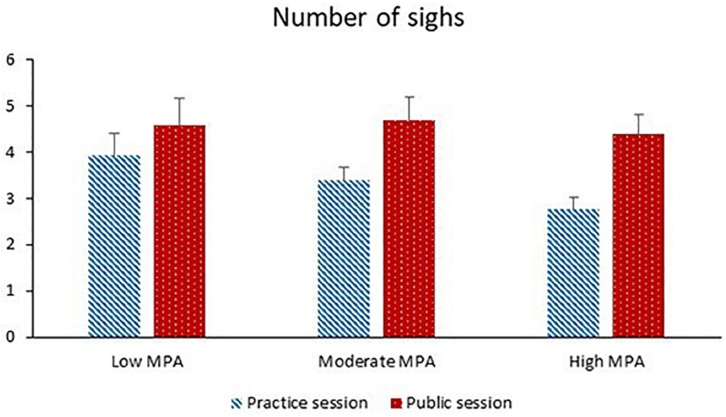
Number of sighs during the practice session and the public session for participants with low MPA (*M* = 34.3, *SD* = 5.0, *n* = 22), moderate MPA (*M* = 46.3, *SD* = 3.0, *n* = 21), and high MPA (*M* = 58.7, *SD* = 6.1, *n* = 22).

The participants exhibited significantly more sighs during the public session than the practice session. Higher motion was significantly associated with more sighing. Session and MPA significantly interacted with each other (see Figure 3). The session effect was significant for MPA levels higher than 45 (*p*s ≤ 0.001–0.006). The MPA effect was not significant for either the practice session (*p* = 0.16) or the public session (*p* = 0.88).

#### Respiratory Means

The factor analyses revealed that mean V’_E_ and mean V_T_ could be tested together as repeated measures of the same latent variable, whereas mean T_I_ and mean T_E_ had to be analyzed separately. Mean V’_E_ and mean V_T_ were significantly higher during the public session than the practice session, were significantly lower during the post-performance phase than the pre-performance phase and significantly increased with increasing motion. Session and phase interacted significantly with each other. The session effect was significant for the pre-performance phase (*p* < 0.001) and non-significant for the post-performance phase (*p* = 0.13). The phase effect was significant for the public session (*p* < 0.001) and non-significant for the practice session (*p* = 0.096).

Mean T_I_ was significantly shorter during the post-performance phase than the pre-performance phase. Higher BMI was significantly associated with longer mean T_I_. Session and phase significantly interacted with each other. During the pre-performance phase, mean T_I_ was longer during the public session than the practice session (*p* = 0.036). During the post-performance phase, mean T_I_ was shorter during the public session than the practice session (*p* < 0.001). Mean T_I_ decreased significantly from the pre-performance phase to the post-performance phase both during the practice session (*p* = 0.004) and the public session (*p* < 0.001). Phase and MPA significantly interacted with each other. The phase effect was significant for MPA levels lower than 60 (*p*s ≤ 0.001–0.020). The MPA effect was not significant for either the pre-performance phase (*p* = 0.70) or the post-performance phase (*p* = 0.63).

For mean T_E_, phase and MPA interacted significantly with each other. Mean T_E_ was shorter during the post-performance phase than the pre-performance phase for MPA levels lower than 40 (*p*s = 0.008–0.013). The MPA effect was not significant for either the practice session (*p* = 0.21) or the public session (*p* = 0.58).

#### Affective State: Anxiety and Tension

**FIGURE 4:**
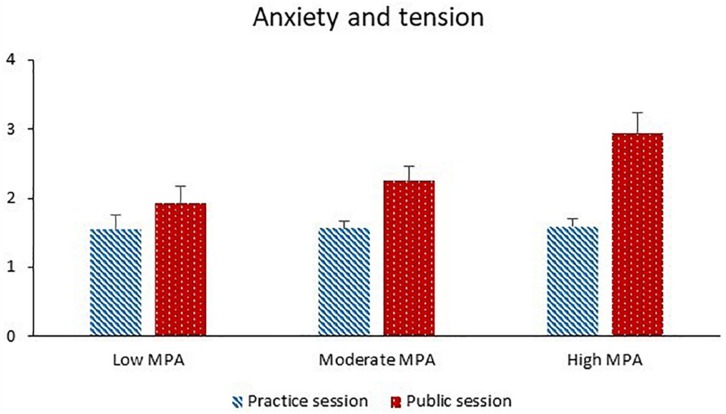
Anxiety and tension during the practice session and the public session for participants with low MPA (*M* = 34.3, *SD* = 5.0, *n* = 22), moderate MPA (*M* = 46.3, *SD* = 3.0, *n* = 21), and high MPA (*M* = 58.7, *SD* = 6.1, *n* = 22). Anxiety and tension were rated on 11-point Likert scales. Higher values correspond to more anxiety and tension.

The factor analysis revealed that ratings of anxiety and ratings of tension could be tested together as repeated measures of the same latent variable “affective state.” Anxiety and tension were significantly greater during the public session than the practice session and before the performance than after the performance. Both higher MPA and higher TA were significantly associated with greater anxiety and tension. Session and phase interacted significantly with each other. The session effect was larger before the performance than after the performance, and the phase effect was larger during the public session than the practice session. Session and MPA significantly interacted with each other (see Figure 4). The session effect increased with increasing MPA, and the MPA effect was significant for the public session (*p* = 0.004) and non-significant for the practice session (*p* = 0.42).

#### Breathing Symptoms

**FIGURE 5:**
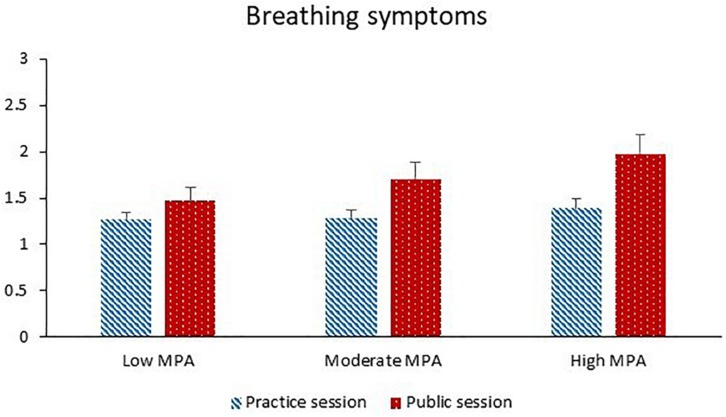
Breathing symptoms during the practice session and the public session for participants with low MPA (*M* = 34.3, *SD* = 5.0, *n* = 22), moderate MPA (*M* = 46.3, *SD* = 3.0, *n* = 21), and high MPA (*M* = 58.7, *SD* = 6.1, *n* = 22). Breathing symptoms were rated on 11-point Likert scales. Higher values correspond to greater shortness of breath and difficulty in breathing deeply enough.

The factor analysis of ratings of shortness of breath and ratings of difficulty in breathing deeply enough revealed that these variables could be tested together as repeated measures of the same latent variable “breathing symptoms.” The participants reported significantly more breathing symptoms during the public session than the practice session, and before the performance than after the performance. Higher levels of TA were significantly associated with more breathing symptoms. Session and MPA significantly interacted with each other (see Figure 5). The session effect increased with increasing MPA, and the MPA effect was significant for the public session (*p* = 0.029) and non-significant for the practice session (*p* = 0.91).

### Relationships Between Self-Reported Measures and Respiratory Measures

Tables 7, 8 show the results regarding the relationships between self-reported measures and respiratory measures. At the within-person level, greater anxiety and tension were significantly associated with higher CV including and excluding sighs, lower AR(1) of V’_E_, higher mean V’_E_ and mean V_T_, longer mean T_I_, and more sighing. There were no significant associations between affective state and respiratory measures at the between-person level. At the within-person level, more breathing symptoms were significantly associated with higher CV including and excluding sighs, lower AR(1) of V’_E_, higher mean V’_E_ and mean V_T_, longer mean T_I_, and longer mean T_E_. At the between-person level, more breathing symptoms were significantly associated with more sighing.

**TABLE 7:** Relationships between self-reported measures, CVs including and excluding sighs, and ARs(1).

	CV including sighs		CV excluding sighs		AR(1) of V’_E_		AR(1) of V_T_		AR(1) of T_I_		AR(1) of T_E_	
						
	Coeff.	*SE*	Coeff.	*SE*	Coeff.	*SE*	Coeff.	*SE*	Coeff.	*SE*	Coeff.	*SE*
**Affective state**												
Between-person component	0.078	0.041	0.056	0.035	**−**0.023	0.017	**−**0.023	0.012	0.011	0.010	**−**0.016	0.015
Within-person component	0.025*	0.012	0.025*	0.011	−0.019**	0.007	**−**0.001	0.006	0.009	0.007	0.001	0.007
**Breathing symptoms**												
Between-person component	0.080	0.060	0.039	0.053	**−**0.009	0.024	**−**0.013	0.018	0.008	0.015	**−**0.013	0.021
Within-person component	0.044*	0.020	0.037*	0.018	−0.038**	0.011	**−**0.000	0.010	0.011	0.012	0.001	0.012

CV, coefficient of variation; AR(1), autocorrelation at one breath lag; V’_*E*_, minute ventilation; V_*T*_, tidal volume; T_*I*_, inspiratory time; T_*E*_, expiratory time; Coeff., estimated coefficient; *SE*, standard error. Statistically significant results are marked in bold. ** *p* < 0.01, * *p* < 0.05.

**TABLE 8:** Relationships between self-reported measures, number of sighs, and respiratory means.

	Mean of V’_E_ and V_T_		Mean of T_I_		Mean of T_E_		Number of sighs	
				
	Coeff.	*SE*	Coeff.	*SE*	Coeff.	*SE*	IRR	95% CI
**Affective state**								
Between-person component	−0.257	0.143	0.002	0.024	0.018	0.033	1.15	0.98–1.35
Within-person component	0.043**	0.015	0.018**	0.006	0.012	0.008	1.07*	1.02–1.12
**Breathing symptoms**								
Between-person component	−0.176	0.211	0.039	0.034	−0.023	0.048	1.33*	1.06–1.67
Within-person component	0.080**	0.025	0.023*	0.010	0.026*	0.013	1.08	0.99–1.18

V’_*E*_, minute ventilation; V_*T*_, tidal volume; T_*I*_, inspiratory time; T_*E*_, expiratory time; Coeff., estimated coefficient; *SE*, standard error; IRR, incidence-rate ratio. Statistically significant results are marked in bold. ** *p* < 0.01, * *p* < 0.05.

## Discussion

### Effects of Session, Phase, and MPA

The participants exhibited significantly greater CVs, lower AR(1) of V’_E_, and more frequent sighing and reported significantly more anxiety, tension, and breathing symptoms during the public session than the practice session (hypothesis 1). The difference between practice and public session in CVs, sighing, anxiety, tension, and breathing symptoms was larger for participants with higher than lower MPA (hypothesis 4). These findings suggest that the presence of an audience increases irregularity in the respiratory system and more so in individuals reporting relatively higher than lower MPA. These findings are compatible with the idea that performing in front of an audience represents a social-evaluative stressor that elicits greater perceived threat of the social self than performing without an audience present, and that the increase in perceived threat from a practice to a public performance is greater for participants reporting relatively higher than lower MPA.

Only anxiety, tension, and breathing symptoms were greater for the pre-performance phase than for the post-performance phase (hypothesis 2), and only for anxiety and tension was the session effect larger during the pre-performance phase than during the post-performance phase (hypothesis 3). We had predicted that the effect of session (i.e. practice vs. public) on respiratory variability and sighing would be more evident prior to performing than after performing. Contrary to this hypothesis, AR(1) of T_I_ and AR(1) of T_E_ were significantly lower during the post-performance phase than the pre-performance phase of the public session. These findings suggest that stability in the respiratory time parameters decreased during the post-performance phase compared to the pre-performance phase of the public session. Future studies should analyze longer post-performance phases to determine how long this increased respiratory instability persists.

Greater MPA was associated with lower CVs. This negative association is noteworthy considering that TA was positively associated with CVs. TA was also positively associated with the affective state. Thus, whereas TA was positively associated with both the affective state and CVs, MPA was positively associated with the affective state but negatively associated with CVs. Two studies have reported similar negative associations between respiratory variability and personal characteristics that share some degree of conceptual similarity with MPA; negative fear of failure (Van den Wittenboer et al., 2003) and negative affect (Van Diest et al., 2006).

Reduced tonic variability in bodily systems (e.g. heart, brain, endocrine) has been generally regarded as a sign of reduced flexibility to generate new patterns in response to changing demands and has been associated with ill-health and aging (Pool, 1989; Lipsitz, 2004; Beauchaine and Thayer, 2015). As there was no significant main effect of MPA on any ARs(1) variables, we could tentatively interpret the negative association between MPA and CVs as a sign of tonically lower sensitivity of the respiratory system in music students with higher than lower MPA (Vlemincx et al., 2013a). Ambulatory monitoring studies are needed to investigate whether this extends beyond performance situations. These studies may also include other psychophysiological measures in order to determine whether students with low and high MPA differ in their tonic levels of other physiological systems. An ambulatory monitoring study with an independent sample of music students found that greater MPA was significantly associated with lower salivary cortisol level during a 7-day period that included a solo performance (Gomez et al., 2018). A link has been shown between activation of the hypothalamic-pituitary-adrenal axis and respiratory irregularity (Abelson et al., 2008).

### Coupling Between Experiential and Respiratory Responses

At the within-individual level, greater anxiety, tension, and breathing symptoms were associated with greater CVs and lower AR(1) of V’_E_. Anxiety and tension were significantly associated with more frequent sighing. These findings extend the results of previous studies in which the associations between affective responses and respiratory responses could only be inferred indirectly (Boiten, 1998; Blechert et al., 2006; Rainville et al., 2006; Vlemincx et al., 2012, 2013b, 2015). Ayala et al. (2010) correlated changes from neutral to unpleasant film clips in self-reported anxiety, breathing symptoms, and tidal volume variability in blood phobic patients. Their findings were partly in line with ours as they found changes in tidal volume variability to be correlated positively with changes in breathing symptoms.

In addition to these anticipated associations involving respiratory variability and sighing, we also obtained significant relationships between the self-reported measures and the means of respiratory parameters. Greater anxiety, tension, and breathing symptoms were significantly associated with greater mean of V’_E_, greater mean of V_T_, and greater mean of T_I_ (and for breathing symptoms also greater mean of T_E_). Ayala et al. (2010) also obtained positive correlations between anxiety, mean of V’_E_ and mean of V_T_ and between shortness of breath and mean of V’_E_, mean of V_T_, and total breath duration. Yet, results of other studies suggest that anxiety may be associated with faster and shallower breathing rather than slower and deeper breathing (see reviews by Boiten et al., 1994; Kreibig, 2010). For instance, Blechert et al. (2006) observed that respiratory rate increased and mean V_T_ decreased from a baseline phase to a threat of shock phase intended to induce anxiety in healthy individuals. Elucidating the origin of these contrasting findings in the context of anxiogenic situations is an important goal to pursue in future work.

At the between-individual level, we found only one significant association. Compared to participants who reported fewer breathing symptoms, participants who reported more breathing symptoms also exhibited more frequent sighing. This lack of significant associations at the between-person level is noteworthy considering that we had a relatively large sample compared to the sample size of most previous studies in the domain of music performance and that we controlled for sources of between-person variance. This dearth of significant associations at the between-person level joins a long list of null results in the domain of music performance (see section “Introduction”).

Our findings suggest that increasing levels of anxiety and breathing symptoms are accompanied by increasing randomness and instability in the respiratory system (Vlemincx et al., 2013a). Indirectly, they are also compatible with the proposition that sighing serves psychophysiological regulatory functions under stress situations that induce negative emotions and negative bodily experiences (Vlemincx et al., 2013a, 2018). Sighing was shown to reset non-random respiratory variability when breathing becomes increasingly random, to be followed by increased stress relief and decreased muscle tension (Baldwin et al., 2004; Vlemincx et al., 2010a, 2016), and to be enhanced during dyspnea relief (Vlemincx et al., 2018). A neurophysiological mechanism put forward to explain the link between anxiety/fear and sighing relates to the generation of sigh in the pre-Bötzinger complex, which is modulated by several brain structures involved in defensive behavior (Davenport et al., 2004; Ramirez, 2014).

These results point to the importance of a within-individual analytical approach. Knowing that a musician experiences higher or lower levels of anxiety or breathing symptoms than another musician in a specific performance situation does not allow us to make meaningful predictions about their breathing patterns. Similarly, information about their breathing patterns will not permit any inferences about what they are experiencing.

The positive within-individual association of CVs with momentary anxiety and tension (i.e. state) contrasts with the negative association of CVs with participants’ general MPA level (i.e. trait). One should not assume that an effect that is true at the state level is also true at the trait level, and vice versa.

### Limitations and Outlook

The order of the two performance sessions mirrored real performance situations as best as possible, that is, practice followed by concert. Because the participants attended a familiarization session, novelty was not an issue in the practice session. The participants were familiar with performance situations so that no habituation was to be expected during the study period.

We measured MPA with the STAI. Other questionnaires can be used to measure MPA such as the Kenny-Music Performance Anxiety Inventory (Kenny, 2011) or the Performance Anxiety Questionnaire (Cox and Kenardy, 1993). These questionnaires are based on different anxiety models and do not highly correlate with each other. Consequently, results of studies using these other questionnaires might differ from those reported here.

The design of the present study included two different performance situations (practice and public). Future researchers may use study designs with more diverse performance situations, in which factors expected to influence perceived threat of the social self are manipulated (solo vs. ensemble performances, small vs. large audiences, audition vs. exam vs. concert, etc.). This way response system coherence could be tested based on a larger number of observations and on a larger range of response magnitude per musician than in this study.

The present investigation focused on self-reported anxiety, breathing symptoms, and respiratory activity. Research should be extended to other measures in order to determine to what extent the associations (and lack thereof) observed in this study between self-reported measures and respiratory responses extend to other physiological responses. Based on the dual-process framework of response system coherence (Evers et al., 2014), one may, for instance, predict that associations between self-reported experience and cardiovascular or endocrine measures are more modest than those obtained for the respiratory system.

Depressive symptomatology and musculoskeletal pain have relatively high prevalence among musicians (Hildebrandt et al., 2012; Kenny et al., 2014; Kenny and Ackermann, 2015), and sighing frequency has been linked with both depression and pain (Keefe and Block, 1982; Boiten, 1998; Robbins et al., 2011). Future work on respiratory psychophysiology in musicians may refine the picture by taking into account these factors.

Future studies should build on this work and explore the potential (long-term) implications of the present results. One line of investigation relates to performance quality. Breathing regulation has been associated with cognitive and motor performance (Marangoni and Hurford, 1990; Karavidas et al., 2010; Grassmann et al., 2016). One mechanism through which specific alterations in the breathing pattern might affect music performance quality is by altering the partial pressure of carbon dioxide (pCO_2_). Subtle perturbations of normal breathing characterized by sighing and increased total variability can lead to lower arterial pCO_2_ (Wilhelm et al., 2001; Alpers et al., 2005; Vlemincx et al., 2013a), which could negatively affect motor and cognitive performance. Sighing, altered respiratory variability, and their associated breathing symptoms could also affect performance outcomes negatively by diverting musicians’ attention from the task at hand. This shift of attention to one’s own breathing may also lead to maladaptive cognitions (e.g. worries, catastrophizing) which may further contribute to performance decrements. Musicians report to focus on breathing frequently and to have respiration-related thoughts (e.g. “Breathe out”) when playing under pressure (Buma et al., 2015; Oudejans et al., 2017). Focus on physical aspects, including breathing, is rated important by the musicians to maintain a high level of performance (Buma et al., 2015).

Another line of research relates to the implications of the present findings for musicians’ well-being and health. For instance, the positive changes generally associated with sighing may act as reinforcers, and sighing may become an emotion regulation mechanism to cope with anxiogenic situations. However, this can bear the risk of “excessive” sighing that can override metabolic needs and lead to reduction in pCO_2_ below optimal levels. Sighing also affects frequency oscillations in multiple cardiovascular parameters (Vaschillo et al., 2015); the implications of these effects remain to be explored. Addressing such questions has the potential to help us qualify the observed respiratory patterns as either detrimental or beneficial for the musicians. Giving that breathing is a possible target for intervention, this knowledge would be crucial for guiding the development and implementation of interventions aiming at managing performance-related stress, optimizing performance, and preserving musicians’ well-being and health (van Dixhoorn, 2007; Wells et al., 2012).

## Conclusion

We found that during the 10 min before and after performing in front of an audience, the music students exhibited greater CVs, lower AR(1) of V’_E_, and more frequent sighing and experienced more anxiety, tension, and breathing symptoms than during the 10 min before and after performing alone. For participants reporting relatively higher MPA, the difference between the practice session and the public session in CVs, sighing, anxiety, tension, and breathing symptoms was larger than for participants reporting lower MPA. Across sessions, greater MPA levels were associated with lower CVs. The significant effect of MPA on CVs and number of sighs was not observed for the respiratory means. We conclude that measures of respiratory variability and sighing are sensitive to the presence/absence of an audience and to participants’ MPA level.

Moreover, we found that increasing anxiety, tension, and breathing symptoms were associated intra-individually with deeper and slower breathing, greater CVs, lower AR(1) of V’_E_, and more sighing suggesting relatively good response system coherence between affective experience, breathing-related sensations, and respiratory responses in the context of music performance situations. In contrast, at the between-individual level the only significant association was between breathing symptoms and sighing.

The present findings suggest that psychophysiological monitoring and analysis incorporating measures of respiratory variability and sighing could add a valuable dimension to the understanding of music performance anxiety, the diagnostic decision process, and the intervention outcome assessment.

## Data Availability Statement

The raw data supporting the conclusions of this manuscript will be made available by the authors, without undue reservation, to any qualified researcher.

## Ethics Statement

The studies involving human participants were reviewed and approved by Ethics committee of the canton of Vaud, Switzerland. The participants provided their written informed consent to participate in this study.

## Author Contributions

All authors listed have made a substantial, direct and intellectual contribution to the work, and approved it for publication.

## Conflict of Interest

The authors declare that the research was conducted in the absence of any commercial or financial relationships that could be construed as a potential conflict of interest.

## References

[B1] Abelson J. L. Khan S. Lyubkin M. Giardino N. (2008). Respiratory irregularity and stress hormones in panic disorder: exploring potential linkages. *Depress. Anxiety* 25 885–887. 10.1002/da.20317 17557312

[B2] Abelson J. L. Weg J. G. Nesse R. M. Curtis G. C. (2001). Persistent respiratory irregularity in patients with panic disorder. *Biol. Psychiatry* 49 588–595. 10.1016/S0006-3223(00)01078-7 11297716

[B3] Alpers G. W. Wilhelm F. H. Roth W. T. (2005). Psychophysiological assessment during exposure in driving phobic patients. *J. Abnorm. Psychol.* 114 126–139. 10.1037/0021-843X.114.1.126 15709819

[B4] Aufegger L. Wasley D. (2018). Salivary cortisol and alpha-amylase are modulated by the time and context of musical performance. *Int J. Stress Manag.* 25 81–93. 10.1037/str0000079

[B5] Ayala E. S. Meuret A. E. Ritz T. (2010). Confrontation with blood and disgust stimuli precipitates respiratory dysregulation in blood–injection–injury phobia. *Biol. Psychol.* 84 88–97. 10.1016/j.biopsycho.2010.02.004 20167246

[B6] Baldwin D. N. Suki B. Pillow J. J. Roiha H. L. Minocchieri S. Frey U. (2004). Effect of sighs on breathing memory and dynamics in healthy infants. *J. Appl. Physiol.* 97 1830–1839. 10.1152/japplphysiol.00298.2004 15208293

[B7] Beauchaine T. P. Thayer J. F. (2015). Heart rate variability as a transdiagnostic biomarker of psychopathology. *Int. J. Psychophysiol.* 98 338–350. 10.1016/j.ijpsycho.2015.08.004 26272488

[B8] Blechert J. Lajtman M. Michael T. Margraf J. Wilhelm F. H. (2006). Identifying anxiety states using broad sampling and advanced processing of peripheral physiological information. *Biomed. Sci. Instrum.* 42 136–141. 16817598

[B9] Blechert J. Michael T. Grossman P. Lajtman M. Wilhelm F. H. (2007). Autonomic and respiratory characteristics of posttraumatic stress disorder and panic disorder. *Psychosom. Med.* 69 935–943. 10.1097/PSY.0b013e31815a8f6b 17991823

[B10] Boiten F. A. (1998). The effects of emotional behaviour on components of the respiratory cycle. *Biol. Psychol.* 49 29–51. 10.1016/S0301-0511(98)00025-8 9792483

[B11] Boiten F. A. Frijda N. H. Wientjes C. J. (1994). Emotions and respiratory patterns: review and critical analysis. *Int. J. Psychophysiol.* 17 103–128. 10.1016/0167-8760(94)90027-2 7995774

[B12] Bruce E. N. (1996). Temporal variations in the pattern of breathing. *J. Appl. Physiol.* 80 1079–1087. 10.1152/jappl.1996.80.4.1079 8926229

[B13] Bruce E. N. Daubenspeck J. A. (1995). “Mechanisms and analysis of ventilatory stability,” in *Regulation of Breathing* eds Dempsey J. A. Pack A. I. (New York, NY: Marcel Dekker), 285–313.

[B14] Buma L. A. Bakker F. C. Oudejans R. R. (2015). Exploring the thoughts and focus of attention of elite musicians under pressure. *Psychol. of Music* 43 459–472. 10.1177/0305735613517285

[B15] Cherniack N. S. Voneuler C. Glogowska M. Homma I. (1981). Characteristics and rate of occurrence of spontaneous and provoked augmented breaths. *Acta Physiol,. Scand.* 111 349–360. 10.1371/journal.pone.0176023 6797251

[B16] Cox W. J. Kenardy J. (1993). Performance anxiety, social phobia, and setting effects in instrumental music students. *J. Anxiety Disord.* 7 49–60. 10.1016/0887-6185(93)90020-L

[B17] Craske M. G. Craig K. D. (1984). Musical performance anxiety: the three-systems model and self-efficacy theory. *Behav. Res. Ther.* 22 267–280. 10.1016/0005-7967(84)90007-X 6466277

[B18] Davenport P. W. Shahan C. P. Zhang W. (2004). “Dorsal periaqueductal gray (dPAG) neural activation increases respiratory activity and incidence of augmented breaths,” in *Poster Presented at the 33rd Meeting of the Society of Neuroscience* San Diego, CA, 23–27.

[B19] Ekman P. (1992). An argument for basic emotions. *Cogn. Emot.* 6 169–200. 10.1080/02699939208411068 29100796

[B20] Evers C. Hopp H. Gross J. J. Fischer A. H. Manstead A. S. Mauss I. B. (2014). Emotion response coherence: a dual-process perspective. *Biol. Psychol.* 98 43–49. 10.1016/j.biopsycho.2013.11.003 24239595

[B21] Fancourt D. Aufegger L. Williamon A. (2015). Low-stress and high-stress singing have contrasting effects on glucocorticoid response. *Front.Psychol.* 6:1242. 10.3389/fpsyg.2015.01242 26388794 PMC4559645

[B22] Fernholz I. Mumm J. L. M. Plag J. Noeres K. Rotter G. Willich S. N. (2019). Performance anxiety in professional musicians: a systematic review on prevalence, risk factors and clinical treatment effects. *Psychol. Med.* 49 2287–2306. 10.1017/S0033291719001910 31474244

[B23] Fredrikson M. Gunnarsson R. (1992). Psychobiology of stage fright: the effect of public performance on neuroendocrine, cardiovascular and subjective reactions. *Biol. Psychol.* 33 51–61. 10.1016/0301-0511(92)90005-F 1599999

[B24] Gill A. Murphy F. Rickard N. S. (2006). A preliminary examination of the roles of perceived control, cortisol and perceptions of anxiety in music performance. *Aust. J. Music Educ.* 2006 32–47.

[B25] Gomez P. Nielsen C. Studer R. K. Hildebrandt H. Klumb P. L. Nater U. M. (2018). Prolonged performance-related neuroendocrine activation and perseverative cognition in low-and high-anxious university music students. *Psychoneuroendocrinology* 95 18–27. 10.1016/j.psyneuen.2018.05.018 29787957

[B26] Grassmann M. Vlemincx E. von Leupoldt A. Van den Bergh O. (2016). The role of respiratory measures to assess mental load in pilot selection. *Ergonomics* 59 745–753. 10.1080/00140139.2015.1090019 26444137

[B27] Hildebrandt H. Nübling M. Candia V. (2012). Increment of fatigue, depression, and stage fright during the first year of high-level education in music students. *Med. Probl. Perform. Art.* 27 43–48. 22543322

[B28] Karavidas M. K. Lehrer P. M. Lu S. E. Vaschillo E. Vaschillo B. Cheng A. (2010). The effects of workload on respiratory variables in simulated flight: a preliminary study. *Biol. Psychol.* 84 157–160. 10.1016/j.biopsycho.2009.12.009 20064581

[B29] Keefe F. J. Block A. R. (1982). Development of an observation method for assessing pain behavior in chronic low back pain patients. *Behavi. Ther.* 13 363–375. 10.1016/S0005-7894(82)80001-4

[B30] Kemeny M. E. (2009). Psychobiological responses to social threat: evolution of a psychological model in psychoneuroimmunology. *Brain Behav.Immun.* 23 1–9. 10.1016/j.bbi.2008.08.008 18809488

[B31] Kenny D. Ackermann B. (2015). Performance-related musculoskeletal pain, depression and music performance anxiety in professional orchestral musicians: a population study. *Psychol. Music* 43 43–60. 10.1177/0305735613493953

[B32] Kenny D. Driscoll T. Ackermann B. (2014). Psychological well-being in professional orchestral musicians in Australia: a descriptive population study. *Psychol. Music* 42 210–232. 10.1177/0305735612463950

[B33] Kenny D. T. (2010). “The role of negative emotions in performance anxiety,” in *Handbook of Music and Emotion: Theory, Research, Applications* eds Juslin P. P. N. Juslin P. N. Sloboda J. A. Sloboda P. J. (Oxford: OUP Oxford), 425–451. 10.1093/acprof:oso/9780199230143.003.0016

[B34] Kenny D. T. (2011). *The Psychology of Music Performance Anxiety.* New York, NY: Oxford University Press.

[B35] Kreibig S. D. (2010). Autonomic nervous system activity in emotion: a review. *Biol. Psychol.* 84 394–421. 10.1016/j.biopsycho.2010.03.010 20371374

[B36] Kusserow M. Candia V. Amft O. Hildebrandt H. Folkers G. Troster G. (2012). Monitoring stage fright outside the laboratory: an example in a professional musician using wearable sensors. *Med. Probl. Perform. Art* 27 21–30. 22543319

[B37] Levenson R. W. (1994). “Human emotions: A functional view,” in *The Nature of Emotion: Fundamental Questions* eds Ekman P. Davidson R. J. (New York, NY: Oxford University Press), 123–126.

[B38] Lipsitz L. A. (2004). Physiological complexity, aging, and the path to frailty. *Sci. Aging Knowledge Environ.* 2004 e16–e16. 10.1126/sageke.2004.16.pe16 15103055

[B39] Marangoni A. H. Hurford D. P. (1990). The effect of varying alveolar carbon dioxide levels on free recall. *Brain Cogn.* 13 77–85. 10.1016/0278-2626(90)90041-L 2112005

[B40] Mauss I. B. Levenson R. W. McCarter L. Wilhelm F. H. Gross J. J. (2005). The tie that binds? Coherence among emotion experience, behavior, and physiology. *Emotion* 5 175–190. 10.1037/1528-3542.5.2.175 15982083

[B41] Nielsen C. Studer R. K. Hildebrandt H. Nater U. M. Wild P. Danuser B. (2018). The relationship between music performance anxiety, subjective performance quality and post-event rumination among music students. *Psychol. Music* 46 136–152. 10.1177/0305735617706539

[B42] Oudejans R. R. Spitse A. Kralt E. Bakker F. C. (2017). Exploring the thoughts and attentional focus of music students under pressure. *Psychol. Music* 45 216–230. 10.1177/0305735616656790

[B43] Pfaltz M. C. Michael T. Grossman P. Blechert J. Wilhelm F. H. (2009). Respiratory pathophysiology of panic disorder: an ambulatory monitoring study. *Psychosom. Med.* 71 869–876. 10.1097/PSY.0b013e3181b492ff 19737858

[B44] Pilger A. Haslacher H. Ponocny-Seliger E. Perkmann T. Böhm K. Budinsky A. (2014). Affective and inflammatory responses among orchestra musicians in performance situation. *Brain Behav.Immun.* 37 23–29. 10.1016/j.bbi.2013.10.018 24513877

[B45] Pool R. (1989). Is it healthy to be chaotic? *Science* 243 604–607. 10.1126/science.2916117 2916117

[B46] Rainville P. Bechara A. Naqvi N. Damasio A. R. (2006). Basic emotions are associated with distinct patterns of cardiorespiratory activity. *Int. J. Psychophysiol.* 61 5–18. 10.1016/j.ijpsycho.2005.10.024 16439033

[B47] Ramirez J. M. (2014). The integrative role of the sigh in psychology, physiology, pathology, and neurobiology. *Prog. Brain Res.* 209 91–129. 10.1016/B978-0-444-63274-6.00006-0 24746045 PMC4427060

[B48] Ritz T. Wilhelm F. H. Meuret A. E. Gerlach A. L. Roth W. T. (2009). Do blood phobia patients hyperventilate during exposure by breathing faster, deeper, or both? *Depress. Anxiety* 26 E60–E67. 10.1002/da.20466 19085969

[B49] Robbins M. L. Mehl M. R. Holleran S. E. Kasle S. (2011). Naturalistically observed sighing and depression in rheumatoid arthritis patients: a preliminary study. *Health Psychol.* 30:129. 10.1037/a0021558 21299301 PMC3059549

[B50] Sackner M. A. Watson H. Belsito A. S. Feinerman D. Suarez M. Gonzalez G. (1989). Calibration of respiratory inductive plethysmograph during natural breathing. *J. Appl. Physiol.* 66 410–420. 10.1152/jappl.1989.66.1.410 2917945

[B51] Soltysik S. Jelen P. (2005). In rats, sighs correlate with relief. *Physiol. Behav.* 85 598–602. 10.1016/j.physbeh.2005.06.008 16038951

[B52] Spielberger C. D. (1983). *STAI State-trait Anxiety Inventory for Adults Form Y: Review Set; Manual, Test, Scoring Key.* Redwood City, CA: Mind Garden, Inc.

[B53] Studer R. K. Danuser B. Hildebrandt H. Arial M. Wild P. Gomez P. (2012). Hyperventilation in anticipatory music performance anxiety. *Psychosom. Med.* 74 773–782. 10.1097/PSY.0b013e31825e3578 22826290

[B54] Studer R. K. Danuser B. Wild P. Hildebrandt H. Gomez P. (2014). Psychophysiological activation during preparation, performance, and recovery in high-and low-anxious music students. *Appl. Psychophysiol. Biofeedback* 39 45–57. 10.1007/s10484-014-9240-2 24477850

[B55] Tobin M. J. Chadha T. S. Jenouri G. Birch S. J. Gazeroglu H. B. Sackner M. A. (1983a). Breathing patterns: 1. Normal subjects. *Chest* 84 202–205. 10.1016/S0012-3692(15)33498-X 6872603

[B56] Tobin M. J. Chadha T. S. Jenouri G. Birch S. J. Gazeroglu H. B. Sackner M. A. (1983b). Breathing patterns: 2. Diseased subjects. *Chest* 84 286–294. 10.1378/chest.84.3.286 6884104

[B57] Van den Wittenboer G. Van Der Wolf K. Van Dixhoorn J. (2003). Respiratory variability and psychological well-being in schoolchildren. *Behav. Modif.* 27 653–670. 10.1177/0145445503256320 14531160

[B58] Van Diest I. Thayer J. F. Vandeputte B. Van de Woestijne K. P. Van den Bergh O. (2006). Anxiety and respiratory variability. *Physiol Behav.* 89 189–195. 10.1016/j.physbeh.2006.05.041 16859718

[B59] van Dixhoorn J. (2007). “Whole-body breathing: a systems perspective on respiratory retraining,” in *Principles and Practice of Stress Management* 3rd Edn, eds Lehrer P. M. Woolfolk R. L. Sime W. E. (New York, NY: Guilford Press), 291–332.

[B60] Vaschillo E. G. Vaschillo B. Buckman J. F. Nguyen-Louie T. Heiss S. Pandina R. J. (2015). The effects of sighing on the cardiovascular system. *Biol. Psychol.* 106 86–95. 10.1016/j.biopsycho.2015.02.007 25720947 PMC4386588

[B61] Vlemincx E. Abelson J. L. Lehrer P. M. Davenport P. W. Van Diest I. Van den Bergh O. (2013a). Respiratory variability and sighing: a psychophysiological reset model. *Biol. Psychol.* 93 24–32. 10.1016/j.biopsycho.2012.12.001 23261937

[B62] Vlemincx E. Meulders M. Abelson J. L. (2017). Sigh rate during emotional transitions: more evidence for a sigh of relief. *Biol. Psychol.* 125 163–172. 10.1016/j.biopsycho.2017.03.005 28315375

[B63] Vlemincx E. Meulders M. Luminet O. (2018). A sigh of relief or a sigh of expected relief: sigh rate in response to dyspnea relief. *Psychophysiology* 55 e12979. 10.1111/psyp.12979 28792624

[B64] Vlemincx E. Taelman J. De Peuter S. Van Diest I. Van Den Bergh O. (2011). Sigh rate and respiratory variability during mental load and sustained attention. *Psychophysiology* 48 117–120. 10.1111/j.1469-8986.2010.01043.x 20536901

[B65] Vlemincx E. Taelman J. Van Diest I. Van den Bergh O. (2010a). Take a deep breath: the relief effect of spontaneous and instructed sighs. *Physiol. Behav.* 101 67–73. 10.1016/j.physbeh.2010.04.015 20417649

[B66] Vlemincx E. Van Diest I. De Peuter S. Bresseleers J. Bogaerts K. Fannes S. (2009). Why do you sigh? Sigh rate during induced stress and relief. *Psychophysiology* 46 1005–1013. 10.1111/j.1469-8986.2009.00842.x 19497009

[B67] Vlemincx E. Van Diest I. Lehrer P. M. Aubert A. E. Van den Bergh O. (2010b). Respiratory variability preceding and following sighs: a resetter hypothesis. *Biol. Psychol.* 84 82–87. 10.1016/j.biopsycho.2009.09.002 19744538

[B68] Vlemincx E. Van Diest I. Van den Bergh O. (2012). A sigh following sustained attention and mental stress: effects on respiratory variability. *Physiol. Behav.* 107 1–6. 10.1016/j.physbeh.2012.05.013 22634279

[B69] Vlemincx E. Van Diest I. Van den Bergh O. (2015). Emotion, sighing, and respiratory variability. *Psychophysiology* 52 657–666. 10.1111/psyp.12396 25524012

[B70] Vlemincx E. Van Diest I. Van den Bergh O. (2016). A sigh of relief or a sigh to relieve: the psychological and physiological relief effect of deep breaths. *Physiol. Behav.* 165 127–135. 10.1016/j.physbeh.2016.07.004 27404329

[B71] Vlemincx E. Vigo D. Vansteenwegen D. Van den Bergh O. Van Diest I. (2013b). Do not worry, be mindful: Effects of induced worry and mindfulness on respiratory variability in a nonanxious population. *Int. J. of Psychophysiol.* 87 147–151. 10.1016/j.ijpsycho.2012.12.002 23266658

[B72] Wasley D. Taylor A. Backx K. Williamon A. (2012). Influence of fitness and physical activity on cardiovascular reactivity to musical performance. *Work* 41 27–32. 10.3233/WOR-2012-1240 22246300

[B73] Wells R. Outhred T. Heathers J. A. Quintana D. S. Kemp A. H. (2012). Matter over mind: a randomised-controlled trial of single-session biofeedback training on performance anxiety and heart rate variability in musicians. *PloS One* 7:e46597. 10.1371/journal.pone.0046597 23056361 PMC3464298

[B74] Widmer S. Conway A. Cohen S. Davies P. (1997). Hyperventilation: a correlate and predictor of debilitating performance anxiety in musicians. *Med. Probl. Performi. Art.* 12 97–106.

[B75] Wilhelm F. H. Trabert W. Roth W. T. (2001). Physiologic instability in panic disorder and generalized anxiety disorder. *Biol. Psychiatry* 49 596–605. 10.1016/s0006-3223(00)01000-3 11297717

